# Supplementary material for: MetaRibo-Seq measures translation in microbiomes
Source: Nat Commun. 2020 Jun 29;11:3268. doi: 10.1038/s41467-020-17081-z (PMC7324362; doi:10.1038/s41467-020-17081-z)
Supplement: Supplementary file 10 — Supplementary Data 7 [file 41467_2020_17081_MOESM10_ESM.zip › File2/Confidence_High_Taxonomy/123459_out.krona.html]

Javascript must be enabled to view this page.

members
magnitude
magnitudeUnassigned
count
unassigned
taxon
rank

123459\_out

2

superkingdom
2
2

1224
1
phylum

class
1236
1

135625
1
order

712
1
family

genus
1
724


SRS143726\_contig\_number\_contig-100\_28068.28068
species
1
726

1
1239
phylum

1
186801
class

order
186802
1

186803
1
family

1
33042
genus

SRS1041140\_contig\_number\_contig-100\_16164.16165
